# “Classics in Spectroscopy”

**DOI:** 10.3797/scipharm.br-10-03

**Published:** 2010-09-30

**Authors:** Liselotte Krenn

**Affiliations:** Department of Pharmacognosy, University of Vienna, Austria

This is an amazing textbook, fascinating for students who want to learn about the isolation and structure elucidation of natural compounds, but also for experienced chemists and pharmacist as well as for lecturers dealing with this topic. It focuses on the isolation and characterization of 30 substances, many of them very well known in daily life e. g. nicotine, curcumin, menthol.

The first chapter of each example provides the historical, medicinal and/or economic background for the compound and/or its starting material. This is followed by a short survey of early relevant literature and recent reviews. The detailed part on isolation compiles I) the principle of the isolation, II) the method for the isolation of the crude product and III) the purification of the latter. The comprehensive part on spectroscopy starts with the UV/vis spectrum, in case necessary the CD-spectrum, and the IR-spectrum. Different NMR-spectra are presented and discussed as well as the mass spectra at the end of this part. Finally, the reader can answer several challenging questions concerning the respective substance and its structure elucidation. In all monographs many pictures are visualizing the topics.

The answers to the questions in each monograph can be found in the last chapters of the book.

Additionally, short citations from the Old Testament over Indian fairy tales up to contemporary authors and even the Beatles, added to the respective monographs, open very interesting perspectives, with which many scientist are often not that familiar.

The only criticism concerns the main title of the book “Classics in Spectroscopy”, which is quite misleading. The subtitle “Isolation and Structure Elucidation of Natural Compounds” describes the excellent contents, which are a pleasure to read, much better.

